# Maiden voyage into death: are fisheries affecting seabird juvenile survival during the first days at sea?

**DOI:** 10.1098/rsos.181151

**Published:** 2019-01-30

**Authors:** Isabel Afán, Joan Navarro, David Grémillet, Marta Coll, Manuela G. Forero

**Affiliations:** 1 Estación Biológica de Doñana (EBD-CSIC), Avda. Américo Vespucio 26, Sevilla 41092, Spain; 2 Institut de Ciències del Mar (ICM-CSIC), Passeig Marítim de la Barceloneta, 37-49, 08003 Barcelona, Spain; 3 Centre d'Ecologie Fonctionnelle et Evolutive, UMR 5175, CNRS - Université de Montpellier - Université Paul-Valéry Montpellier - EPHE, Montpellier, France; 4 Percy FitzPatrick Institute, DST/NRF Centre of Excellence, University of Cape Town, Rondesbosch, South Africa

**Keywords:** bycatch, habitat modelling, IUU fisheries, long-distance migration, petrels, satellite telemetry

## Abstract

The study of juvenile migration behaviour of seabird species has been limited so far by the inability to track their movements during long time periods. Foraging and flying skills of young individuals are assumed to be inferior to those of adults, making them more vulnerable during long-distance migrations. In addition to natural oceanographic effects and intrinsic conditions, incidental seabird harvest by human fisheries is one of the main causes of worldwide seabird population declines, and it has been hypothesized that juveniles are particularly vulnerable to bycatch during their first weeks at sea after leaving the nest. We used solar-powered satellite tags to track the at-sea movements of adults and juveniles of Scopoli's shearwater (*Calonectris diomedea*) after the autumn departure from their breeding colony in Chafarinas Islands (southwestern Mediterranean Sea). Eighty per cent of juvenile tags stopped transmitting during the first week at sea, within 50 km of their natal colony, in an area with one of the highest concentrations of fishing activities in the Mediterranean Sea. All adult birds tagged and only 20% of juveniles migrated into the Atlantic and southwards along the coast of West Africa. The two age groups showed different habitat preferences, with juveniles travelling farther from the coast, in windier and less productive waters than adults. We conclude that Scopoli's shearwater juveniles are particularly vulnerable to mortality events, and we highlight that fisheries, along with differential age-related behaviour skills between adults and juveniles, are likely causes of this mortality. Overall, our study highlights the importance of conducting tracking studies during the first stages of juvenile migration.

## 1. Introduction

Seabirds are impacted by breeding habitat destruction, invasive terrestrial species feeding on adults and juveniles, and by interactions with fisheries [1,2]. Fisheries affect seabird populations indirectly through competition for marine prey [3], and directly through incidental mortality on fishing gear [4]. Many of these incidental captures remain undetected, especially in illegal, unreported and unregulated fisheries [5] operating in remote areas visited by seabirds during their feeding and migratory journeys. Notwithstanding this paucity of data, several studies have converged in highlighting seabird bycatch as an important factor in many population declines [6].

Incidental mortality is especially relevant for long-lived pelagic seabirds, such as Procellariforms [2]. Mortality associated with fishing affects all age classes; however, juveniles are considered more vulnerable. Juvenile mortality has been associated with inferior foraging skills compared to adults, and with lower levels of individual experience in navigation strategies [7,8]. Differences in foraging grounds could thereby increase juvenile susceptibility to fishing gear [9]. Consequently, the first months of independence are particularly challenging for their survival [10].

Despite major recent advances, studying at-sea movements of juvenile pelagic seabirds remains particularly challenging due to logistical constraints because they are distributed remotely in the open ocean during several years [7]. Specifically, no study has compared at-sea movements and fates of adult and juvenile seabirds while assessing the magnitude of fishing operations within their home ranges. Here we examine the spatial movements of ‘naive’ juvenile Scopoli's shearwater (*Calonectris diomedea*) during their first months at sea using miniaturized satellite-tracking devices. We also fitted tags onto adult birds from the same breeding locality. We then modelled the at-sea habitat selection of adults and juveniles in relation to environmental data, as well as information concerning fishing activities. We addressed two main questions: (i) Do individuals of different age classes show similar migration routes and habitat preferences? and (ii) Are there differences in apparent survival between shearwater age classes?

## 2. Material and methods

### 2.1. Study area and fieldwork procedures

**Figure 1.**
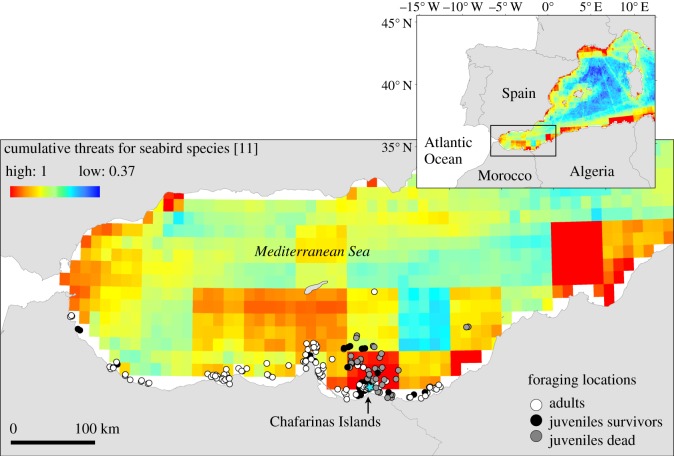
Foraging locations of adults (*n* = 5, white points), juveniles that stopped transmitting during the first 6 days of fledging (*n* = 7, grey points) and juveniles that crossed to the Atlantic Ocean (*n* = 2, black points). Background shows areas of cumulative threats with potential impact on seabirds from Coll *et al*. [11].

This study was conducted at the Chafarinas Islands in the southwestern Mediterranean Sea (figure 1). This small archipelago holds 800 breeding pairs of Scopoli's shearwaters in a highly productive zone supporting a high volume of fisheries [12], mostly artisanal or illegal and not tracked through the Automatic Identification System (AIS) or Vessel Monitoring System (VMS) for monitoring fishing vessels activities. The Scopoli's shearwater is a long-lived seabird with delayed maturity [13]. Immatures only return to the colony when 5 or 6 years old, and the maximum probability of first breeding is reached at the age of seven [14]. Previous studies at Chafarinas Islands showed a diet composed mainly of small pelagic fish and demersal resources present in the bycatch discarded by fisheries into the sea [15].

Tracking devices were solar-powered satellite transmitters (PTTs, platform terminal transmitters, Argos PTT-100, Microwave Telemetry Inc., Columbia, USA). These tags allow seabird positioning through the Argos satellite system. They are an ideal way to follow long-distance migration movements in nearly real time. Since animals are tracked over large spatial scales, devices are difficult to recover [16]. PTTs were deployed during October 2014 (late chick rearing period) on 10 juveniles born during June 2014 and on five breeding adults. Adults and juveniles were captured during the night in their nests. PTTs weighed 18 g (less than 3% of bird body mass of adults = 706 ± 75 g and of juveniles = 785 ± 114 g). PTTs were attached to back feathers using Tesa® tape and Nural92® glue and programmed with a duty cycle of 10 h ON/24 h OFF. PTT data were processed to remove erroneous Argos locations, by using a speed, distance and angle filter procedure with the Argosfilter R package [17], using a maximum threshold speed at 80 km h^−1^ [18]. For each individual, we calculated total distance covered in the Mediterranean and in the Atlantic waters. Distance travelled by day was calculated as total distance (the sum of the distances between each transmission) divided by the range of days between first and last location.

### 2.2. Behavioural modes

Behavioural modes in adult and juvenile individuals were classified with an expectation maximization binary clustering (EMbC) algorithm [19] performed using the EMbC R package [20]. EMbC identifies behavioural modes in movement trajectories based on the velocity and turning angle of a bird between each location in an unsupervised manner. Based on local measures of velocity and turning angle of a bird between each location, locations were classified in four behaviours: (i) resting (low velocity/low turning angle), (ii) intensive search (low velocity/high turning), (iii) extensive search (high velocity/high turning angle) and (iv) travelling (high velocity/low turning angle).

Behavioural modes were compared for each age class among the first days of independence (locations in the Mediterranean Sea) and for the rest of migration time (Atlantic Ocean), by a two-sample test for equality of proportions with continuity correction for small samples.

### 2.3. Human threats

To identify the hazard level of oceanic areas used by Scopoli's shearwaters in the Mediterranean Sea, we used the spatially explicit information of cumulative threats with potential impact on seabirds published by Coll *et al*. [11]. In particular, this index is a weighted sum of each of the following anthropogenic threats affecting seabirds: coastal-based impacts (0.08), marine pollution (0.31), exploitation of resources (0.38), maritime activities (0.08) and climate change (0.15). Vulnerability weights (annotated in parenthesis) applied to seabirds species were estimated using published data on specific taxa and expert opinions (see [11] for details). The index value was then extracted for each foraging location of the individuals in the Mediterranean Sea. Kruskal–Wallis tests were conducted to examine differences in index values at each location associated with differences in age and survival (adults, juveniles that stopped transmitting data, and juveniles that transmitted as far as the Atlantic Ocean). Analyses were performed in R v. 3.4.1 [21].

### 2.4. Environmental variables

The oceanic habitat used by juveniles and adults during their at-sea movements was modelled with habitat suitability models through a maximum entropy approach [22] using chlorophyll-a, oceanic winds and bathymetry to describe the oceanographic habitat. Chlorophyll-a concentration (mg m^−3^), was used as a proxy for primary productivity. Oceanic winds are one of the most important factors affecting migratory behaviour of seabirds, particularly in birds performing dynamic soaring such as shearwaters [23], and bathymetry was used as a proxy of coastal versus pelagic domains. In order to model environmental data consistent with the seabird tracking time period (four months), we divided the latitudinal range of shearwater migratory habitat into different tiles (two time periods for chlorophyll-a data and three time periods for winds data). Chlorophyll-a and wind data were sourced for each tile covering the time range of tracking data within the tile. Tiles were merged into a single raster as input for the species distribution model. To facilitate comparisons across months, we normalized the images to the scale of 0–1, based on the lowest and highest values of any pixel in any of the tiles [2]. Chlorophyll-a (mg m^−3^) data were obtained from aqua MODIS sensor (https://oceancolor.gsfc.nasa.gov/), at a spatial resolution of 0.08333° (approx. 9 km). We downloaded autumn 2014 seasonal composite data for the first temporal tile (which covers tracking data from the first departure, 8 October 2014 until 15 November 2014) and the 2014 wintering seasonal composite for the second temporal tile (time range from 15 November 2014 until 22 January 2015). Bathymetry was downloaded from the Gridded Global Relief Data ETOPO2v2 database (https://www.ngdc.noaa.gov/mgg/global/etopo2.html) at a spatial resolution of 0.033° (approx. 3 km). Wind data were obtained from the NOAA Blended Sea Winds (https://www.ncdc.noaa.gov/data-access/marineocean-data/blended-global/blended-sea-winds), which contain globally gridded, high resolution ocean surface vector winds and wind stresses on a global 0.25° grid. Wind data were disaggregated into their zonal (east–west) and meridional (north–south) components. Data were downloaded daily for the October 2014–January 2015 period, and merged in three temporal tiles according to tracking data (from 8 October 2014 until 24 October 2014, second tile from this date until 2 November 2014 and third tile until the last transmission recovered on 22 January 2015). All data were resampled to 0.25°, the coarser resolution supplied by wind data.

### 2.5. Habitat use

Habitat suitability models were developed through a maximum entropy approach (Maxent) [3,4], which models species distribution by estimating the density of environmental covariates conditioned to a species' presence [4]. We ran separated models for adults and juveniles. Models were constructed with the interface of the standalone Maxent program v. 3.4.0 k (https://biodiversityinformatics.amnh.org/open_source/maxent/). Default parameters were used. Only linear relationships between estimated probabilities of presence and environmental variables were fitted. Model performance was assessed by randomly dividing species occurrence data into training (70%) and test (30%) datasets, by using the option ‘random test percentage’ in Maxent. A given model was calibrated on the training data and evaluated on the test data using the area under the receiver operating characteristics curve (AUC) as a threshold-independent assessment measure. To reduce uncertainty caused by sampling artefacts (generated during the random resampling of presence occurrence localities), we conducted 15 replicate models for each of the four environmental variables (bathymetry, chlorophyll-a, and zonal and meridional components of sea surface winds). We evaluated their contribution to the Maxent model with a jackknife procedure.

## 3. Results

We successfully recovered 2691 at-sea locations (2108 for adults and 583 for juveniles), distributed between 7 October 2014 and 22 January 2015. Adults were tracked for 52–103 days, whereas the tracking period for juvenile birds was much shorter (0–57 days, table 1). Only two of the ten tracked juveniles transmitted more than one week and reached the Atlantic Ocean. The PTT of a juvenile lost during the first day of emission near the colony where most individuals disappeared, was detected after one week of silence near an Algerian harbour, 150 km away (see electronic supplementary material, figure S1). Based on the Coll *et al*. [11] index, high cumulative threats to seabird species were concentrated in their foraging areas within a 50 km radius of the colony, where seven juveniles stopped transmitting (figure 1). PTT-equipped juveniles that failed to emit in the vicinity of the colony showed higher values of the cumulative threat index (mean ± s.d., 0.67 ± 0.2) than survivors (0.42 ± 0.35) and adults (0.43 ± 0.12). Significant differences (*χ*^2^ = 11.43, *p* = 0.003, d.f. = 2) were found among the three categories of age (adults, juveniles that stopped transmitting and juvenile survivors). Nevertheless, differences between groups were only significant for adults and dead juveniles.

**Table 1.** Tracking details of the Argos PTT deployments on Scopoli's shearwaters during the chick-rearing period 2014 in Chafarinas Islands (SW Mediterranean).

individual	age	number of filtered locations	date of first Argos fix	date of last Argos fix	total duration (days)	total distance (km)
6134623	adult	431	11 Oct 2014	17 Dec 2014	67.5	8554.5
6110249	adult	328	7 Oct 2014	3 Dec 2014	57.3	6507.5
6114951	adult	639	11 Oct 2014	22 Jan 2015	103.7	14159.5
NX01444	adult	487	11 Oct 2014	28 Dec 2014	78.3	9216.5
NX01445	adult	319	11 Oct 2014	2 Dec 2014	52.8	8690.7
NX01407	juvenile	33	13 Oct 2014	19 Oct 2014	5.8	143.7
NX01417	juvenile	13	12 Oct 2014	18 Oct 2014	5.4	86.3
NX01419	juvenile	1	10 Oct 2014	10 Oct 2014	0	0
NX01430	juvenile	10	8 Oct 2014	9 Oct 2014	1.7	37.6
NX01433	juvenile	13	9 Oct 2014	15 Oct 2014	5.8	137.5
NX01434	juvenile	8	9 Oct 2014	15 Oct 2014	5.6	119.4
NX01437	juvenile	355	16 Oct 2014	13 Dec 2014	57.8	8192.8
NX01440	juvenile	137	11 Oct 2014	11 Nov 2014	31.1	4463.2
NX01441	juvenile	11	9 Oct 2014	11 Oct 2014	1.7	44.07
NX01442	juvenile	3	11 Oct 2014	11 Oct 2014	0.1	7.6

**Figure 2.**
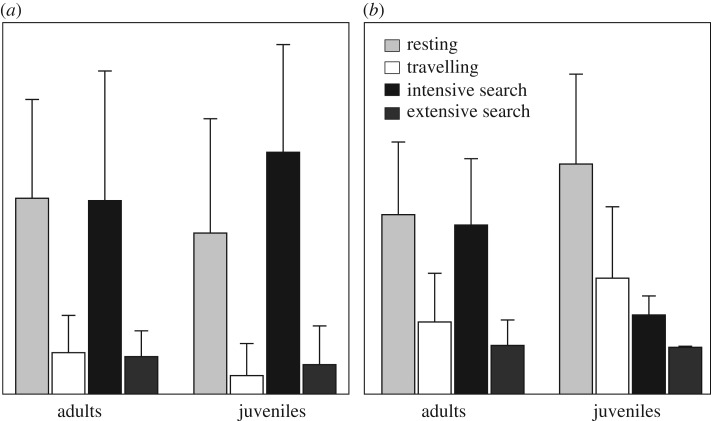
Mean and standard deviation of the different behavioural states of adults and juveniles in (*a*) the Mediterranean Sea and (*b*) the Atlantic Ocean.

Behavioural models (figure 2) showed higher rates of intensive search behaviour in the Mediterranean in juveniles compared to adults, whereas in the Atlantic Ocean, juveniles showed a higher proportion of resting and travelling, and intensive search became the most important behaviour in adults (figure 2). Behavioural models (figure 2) showed significantly higher rates of intensive search (*χ*^2^ = 23.70, *p* < 0.001, d.f. = 1) and travelling behaviour (*χ*^2^ = 15.29, *p* < 0.001, d.f. = 1) in the Mediterranean Sea compared to the Atlantic Ocean for juveniles, whereas adults did not show differences in behaviour strategies throughout the whole monitoring period.

**Figure 3.**
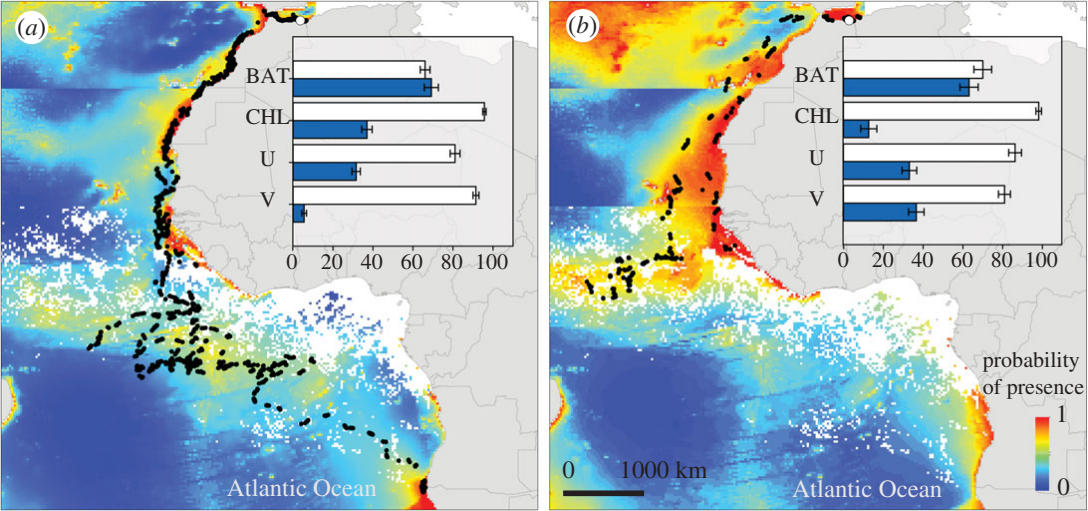
Outputs of the species distribution models for adults (*a*) and juveniles (*b*) of Scopoli's shearwaters during the first three months of wintering migration. Black points show tracked filtered-locations registered by Argos PTT devices. Horizontal colour jumps show the temporal ranges of environmental variables used in this study. The white dot shows colony location (Chafarinas Islands). Bar charts show the relative contribution of explanatory variables considered in the Maxent model. Average and s.d. over replicate runs for the importance of each habitat variable as estimated by the Jackknife test. For each variable, white bar indicates the explanatory power of the model when the single environmental variable is omitted, and lower grey bar indicates the explanatory power (in terms of regularized training gain) of the model when the environmental variable is used in isolation. Variables: BAT—bathymetry, CHL—chlorophyll a, U—zonal wind component, V—meridional wind component.

All five tracked adults reached the Atlantic Ocean, between 16 and 23 October. The only two surviving juveniles left the Mediterranean Sea, crossing into the Atlantic, between 21 and 23 October. Total distance travelled per day was slightly higher in juveniles than in adults in the Atlantic (164.3 ± 18.6 km in juveniles and 142.0 ± 22.9 km in adults), but markedly lower in the Mediterranean (31.4 ± 17.3 km in juveniles versus 88.2 ± 38.9 km in adults). The migration routes of the five adults and of one juvenile were similar, flying parallel to the African coast. In contrast, the second juvenile travelled almost constantly west of the continental shelf (figure 3).

Habitat models showed good ability to predict shearwater distributions during migration (AUC = 0.8 for both juveniles and adults). Bathymetry was the variable with the highest contribution to distribution of shearwaters during migration. Probability of occurrence of juveniles and adults increased in shallow waters. Chlorophyll-a showed a higher explanatory power for adults (37.0%) than juveniles (12.9%). In relation to wind effect, juveniles were more affected by the meridional (north–south direction) component of winds than adults (figure 3).

## 4. Discussion

This study is one of the few describing foraging and migration movements of a marine predator during the first weeks of its life at sea [24,25]. One of the main results is the unexpected loss of communication with 80% of shearwater juveniles within a few days after leaving the nest. Although based on a small sample size (*n* = 10), juvenile tracking failure exceeded those of previous investigations on this species at other Mediterranean colonies. Péron & Grémillet [26] reported that 40% of the juveniles and 34% of the adults tagged (total individuals tagged, *n* = 12 juveniles and *n* = 3 adults) on the Italian Mediterranean coast stopped communicating before crossing into the Atlantic Ocean. Further, Raine *et al.* [27] reported that 66.6% of tracked juveniles (*n* = 3) vanished in the Mediterranean Sea. Since the deployment period and attachment methodology were the same for adults and juveniles and only juveniles were affected, we consider that high PTT communication failure showed in the present study was associated to events affecting mainly juvenile shearwaters.

Although different stressors could explain these observations, such as strong storms [28] or light-induced mortality [29], we argue that mortality associated to fishing activity was one of the potential causes of observed juvenile disappearance [30]. Indeed, no storm was recorded in the area within which juveniles vanished (based on the wind data downloaded from NOAA database, see Material and methods). Also, artificial lights associated to a nearby tourist resort may have attracted satellite-tracked juveniles towards land, with likely casualties [29]; however, this resort was still under construction during the study period, with very limited night-time activities, and we did not record any position on land within this area [31].

In contrast, juvenile tracking failures occurred all at once while birds were displaying intensive foraging behaviour in an area of high cumulative threats for seabirds, mainly from fisheries [11]. Although there is no conclusive evidence, we therefore suggest that juveniles probably could die accidentally in long-line fishing gears, one of the main reported causes of shearwater mortality in the Mediterranean Sea [32]. It is noteworthy that this hazardous area north of the colony has been reported as an illegal swordfish fishing area [33], where the Moroccan and Algerian fleets operate year-round. Furthermore, Morocco has the lowest level of compliance with international fisheries regulation within countries bordering the North Atlantic [34]. Also, one PTT-equipped juvenile that stopped transmission in this area, sent the last two positions close to the Algerian harbour of Bouzedjar, after 6 days without reporting tracking locations (see electronic supplementary material, figure S1). This harbour holds small-scale artisanal fisheries, a type of fishing activity also linked with high seabird bycatch probabilities [30,35], thus we suspect that bird was caught during fishing activities and transmitter released from bird and saved until arrival at the harbour. All of these circumstantial elements point to fisheries exploitation as one of the main potential causes of juvenile loss. When fledging from their natal colony, juvenile seabirds have reduced foraging skills, and face a long period of learning. These first few months are critical in terms of survival [7]. We therefore speculate that, because of their relative handicap, juvenile shearwaters fledging from our study colony initially gathered within a nearby high-productivity area, which should ensure their efficient foraging, but was unfortunately also a potential ‘death zone’ in terms of fisheries bycatch.

Along with threats caused by illegal fishery practices, juvenile behaviour could contribute to high mortality. During their first two weeks in the Mediterranean Sea, juveniles registered more intensive searching behaviour than adults. The breeding colony of Chafarinas Islands is in the vicinity of fishing harbours and intense (mostly illegal) fishing activities [33]. Those generate visual and olfactory cues, which may attract juvenile shearwaters towards fishing vessels, and to an early death. Travelling behaviour gained in importance once juveniles reached the Atlantic, where space use by Scopoli's shearwaters from Chafarinas Islands off West Africa was similar to that of individuals from other Mediterranean colonies [36]. Juvenile tracks were more affected by winds than adults, moving them away from productive shelf waters selected by adults [26]. Such exploitation of different regions could also lead to age-biased exposure to bycatch [37].

Overall, our study suggests that mortality affected juveniles more severely than adults. Illegal fisheries and inefficiency in juvenile foraging skills might be the main causes of juvenile death, but the exact magnitude of each forcing factor remains unknown. From a precautionary point of view, and as highlighted by other studies [38], it seems extremely urgent to detect and control illegal seabird incidental bycatch and intentional harvest, as those illegal practices weigh heavily on the persistence of vulnerable seabird populations at the scale of northwest Africa and of the western Mediterranean.

## Supplementary Material

Figure S1

## Data accessibility

Our data are deposited at Dryad: http://dx.doi.org/10.5061/dryad.h3p550p [39].
