# Supplementary material for: Maiden voyage into death: are fisheries affecting seabird juvenile survival during the first days at sea?
Source: R Soc Open Sci. 2019 Jan 30;6(1):181151. doi: 10.1098/rsos.181151 (PMC6366166; doi:10.1098/rsos.181151)
Supplement: Figure S1 [file rsos181151supp1.pdf]

Maiden voyage into death: are fisheries affecting seabird juvenile survival during the first days at sea?

Isabel Afán, Joan Navarro, David Grémillet, Marta Coll, and Manuela G. Forero

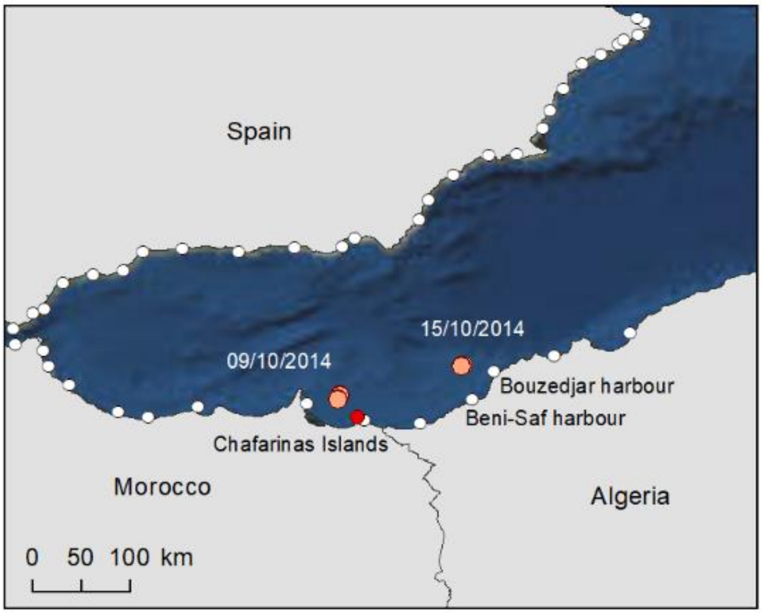

Figure S1. Spatial locations retrieved from one of the Scopoli's shearwater juveniles tracked. This individual emitted information during two different days, with a silent lapsus of five days among with. Between the first and second days of transmission, individual travelled 150 km east, towards a coastal zone in the vicinity of two main Algerian fishery harbors. Chafarinas Islands, the breeding colony, is showed by a red dot. White dots indicate main fishing ports.
